# Incidence and presentation of vestibular schwannoma: a 3-year cohort registry study

**DOI:** 10.1007/s00701-023-05665-9

**Published:** 2023-07-15

**Authors:** Rocio Fernández-Méndez, Yizhou Wan, Patrick Axon, Alexis Joannides

**Affiliations:** Clinical Neurosciences, University of Cambridge, Addenbrooke’s Biomedical Campus, Cambridge, Cambridgshire CB2 0QQ UK

**Keywords:** Vestibular schwannoma, Acoustic neuroma, Epidemiology study, Clinical registry

## Abstract

**Background:**

Vestibular schwannoma (VS) is the most common benign tumour arising in the lateral skull base. Reported incidence rates of VS vary across geographical locations and over time. There is scarce updated evidence over the past decade on the epidemiology and mode of presentation of VS.

**Objective:**

To describe the epidemiology and mode of presentation of VS in the East of England between 2013 and 2016.

**Methods:**

A retrospective epidemiological analysis of data from a national VS registry and electronic patient records was conducted, including all newly diagnosed adult patients in a UK tertiary referral centre, between April 1st, 2013, and March 31st, 2016.

**Results:**

There were 391 new cases identified resulting in an overall mean incidence of 2.2 VS cases per 100,000 person-year. The incidence rate for all patients in the <40 age group ranged between 0.3 and 0.7 per 100,000 person-year, increasing to a range of 5.7 to 6.1 per 100,000 person-year in the 60–69 age group. The top three combinations of symptoms on presentation per patient were hearing loss and tinnitus (97, 24.8%), hearing loss alone (79, 20.2%) and hearing loss, tinnitus, and balance symptoms (61, 15.6%). The median duration of symptoms was 12 months, with a wide range from 1.4 to 300 months. Age was negatively correlated with tumour size (r = -0.14 [-0.24 to -0.04], *p*=0.01) and positively correlated with symptom duration (r = 0.16 [0.03–0.29], *p*=0.02).

**Conclusions:**

The incidence of vestibular schwannoma has increased compared to previous studies in the UK and is similar to incidence rates reported in other countries during the past decade. It peaks in the seventh decade of life, mainly because of an increase in the diagnosis of small tumours with a long duration of audio-vestibular symptoms in older patients, compared to earlier studies.

## Introduction

Vestibular schwannomas (VS) are benign tumours arising from Schwann cells of the eighth cranial nerve [13, 24, 25]. Epidemiological studies have suggested that the incidence of sporadic VS has been increasing globally [11, 15, 17, 18, 22]. In the decade prior to 2010, the incidence of VS was shown to be between 1.0 and 3.7 per 100,000 person-years [10, 11, 17]. A recent systematic review showed that the incidence of VS between the years 2010 and 2020 ranged between 3.0 and 5.2 per 100,000 person-years [15]. This has been largely attributed to improved access to diagnostic imaging, especially MRI [17, 18]. In the UK, mean incidence rates have been reported to range from 1.0 to 1.4 patients per 100,000 persons per year, but these studies are two to three decades’ old [5, 20].

Key context-specific differences, such as funding of health services, population access to MRI scans and demographical characteristics, as well as methodological differences in the identification of cases, may explain the reported variability in incidence rates, and complicates the generalisability of study findings beyond their respective study populations [10, 11, 15, 17, 22]. The use of administrative registries in epidemiology studies has been shown to potentially lead to a systematic underreporting of cases compared to prospectively maintained and standardised disease-specific registries [16, 19]. In particular, standardised reporting of key factors such as tumour size has been recommended to facilitate comparison of diagnostic strategies and treatment outcomes. For example, the Tokyo recommendations on reporting VS size grade tumours in small, medium, moderately large, large and giant tumours based on maximum extrameatal planar measurements [8]. The increased utilisation of MRI scans, particularly in high-income countries, has led to an increased number of smaller tumours being diagnosed in those countries. Compared to larger tumours where surgery is preferred, these tumours pose a particular challenge regarding the optimal strategy for management. Over recent years, there has been a shift towards less invasive management strategies for VS, including conservative management and stereotactic radiosurgery, to maintain quality of life and reduce treatment-associated comorbidity [2, 3].

It is important to understand the incidence of VS across different geographical regions, in the context of different health systems and demography, in order to plan the delivery of diagnostic and treatment services. In countries where services are delivered across multiple geographical regions or where there is centralised planning of specific treatment services such as radiosurgery, an accurate understanding and comparison of the epidemiology of VS across different regions becomes even more relevant to guide planning. Furthermore, understanding the presenting patient characteristics and symptoms alongside the incidence is key to aid with the development of screening strategies for these patients.

In the UK, all VS cases are referred to specialist skull base units for discussion in tumour board meetings about management decisions. England has 21 skull base units, each of which covering a geographically defined population based on their catchment areas. Our skull base unit covers the East of England, and all patients with a radiological diagnosis of VS are prospectively entered into the UK Vestibular Schwannoma Registry (UKVSR). Each patient’s registry record is linked to their electronic medical record (EPR). This offers us a unique opportunity to investigate VS epidemiology and presenting characteristics of VS patients, in a well-defined geographical area. On this background, with this study, we aimed to characterise the incidence of VS, as well as the frequency distribution of presenting symptoms on diagnosis of VS, in the East of England.

## Methods

### Study design

A single-centre retrospective study was conducted based on review of medical records from all patients referred to a tertiary centre covering the population in the East of England, between April 1st, 2013, and March 31st, 2016.

### Participants

All patients, 18 years or older, referred to the study centre and receiving the first diagnosis of unilateral VS were included. Patients who were classified as follow-up cases and patients receiving a diagnosis outside the specified time-period were excluded.

### Data sources

Data sources included the UK Vestibular Schwannoma Registry (UKVSR) and the hospital electronic patient records (EPR) implemented in the study centre. The UKVSR is a national database recording individual-level cases of VS referred to UK skull base centres. It is overseen by the British Skull Base Society and hosted by the ORION health informatics platform (Orion MedTech Ltd CIC; Cambridge, UK). In our centre, all patients who are diagnosed with VS are prospectively recorded in the UKVSR. Data collected from the UKVSR included demographics (age at diagnosis and sex), tumour size and tumour location. Presenting symptoms and symptom duration were retrieved from their EPR implemented in the study Centre, Epic (Epic Systems Corporation; Verona, Wisconsin, USA). Records were linked to the UKVSR via unique anonymised patient-identifiers.

Population estimates for census output areas in the East region of England, from the UK Office of National Statistics (ONS), were used for the calculation of incidence rates [26].

### Outcome measures

Tumour size was classified as intrameatal (IM) and extrameatal (EM). The size of extrameatal tumours was determined as the largest extrameatal diameter by linear measurements. This classification follows the Tokyo recommendations on reporting the size of VS and is in line with the British National Vestibular Schwannoma Audit [1, 8]. Presenting neuroimaging was not available but tumours with extrameatal extension were further categorised according to size into small (up to 10 mm), medium, (>10–20 mm), moderately large (>20–30 mm) or large (>30 mm) tumours depending on their maximal extrameatal diameter [8, 28].

Presenting symptoms and duration of symptoms were ascertained from the clinic record at diagnosis, during the first assessment by a Skull Base Neurosurgeon at the Study Centre. Symptoms were grouped into profiles according to each patient’s presenting combination of symptoms. Symptom profiles presenting in 5% of patients or fewer were classified as “Other”. Where a numerical duration was not recorded, the duration of symptoms was classified into (a) incidental, discovered after an unrelated investigation in asymptomatic patients; (b) emergency, presented with hydrocephalus; (c) sudden, with sudden onset of symptoms; (d) acute, with symptoms for less than four weeks; (e) subacute, with symptoms for four weeks to less than 12 months; (f) chronic, with symptoms for 12 months or more; and (g) unclear duration.

### Statistical analysis

Incidence rates per 100,000 person-years calculated using incident cases of VS as the numerator and age and sex-specific counts of the population of East of England during each study year as the denominator. Incident cases were grouped by age in years (< 40, 40–49, 50–59, 60–69, > 69), sex and by years of diagnosis.

Patient and tumour characteristics were presented as proportions and continuous variables were summarised using their mean and median values, as appropriate. Group comparisons were carried out by year of diagnosis, age group, sex, symptom profile, and duration of pre-diagnosis symptoms. Chi-squared test was used to compare categorical characteristics between groups. Independent samples Kruskal Wallis test of medians was used to compare continuous variables between groups, as appropriate. Statistical significance was set at *p*<0.05. Analyses were conducted using R Statistical Software (v4.2.1; R Core Team 2022).

### Ethical considerations

This study was approved by the Quality Improvement Board of the participating centre as a Service Evaluation. Data was analysed at the aggregate level and all extracted patient-level data was anonymised for the analyses to maintain patient confidentiality.

This paper follows the Strengthening the Reporting of Observational Studies in Epidemiology guidelines.

## Results

### Incidence and patient characteristics

During the three-year period, 391 newly diagnosed patients with VS meeting the inclusion criteria were identified. Annually, the number of new diagnoses decreased from 140 during 2013/14 to 122 in 2015/16 (Table 1). There were practically the same number of females and males (Table 1). Pre-treatment tumour sizes were available for all patients except one. Small tumours were generally more common than other tumour sizes across the 3-year period, with large being relatively uncommon (Table 1).

**Table 1:** Characteristics of patients and incidence of diagnosis of vestibular schwannoma, overall and by year of diagnosis

	Overall	By year		
	(Over the 3 years)	2013/14	2014/15	2015/16
No. of patients	391	140	129	122
Age (years), median (IQR)	61.0 (19)	59.5 (19.2)	63.0 (18.0)	60.0 (18.8)
Female sex, n (%)	196 (50.1)	58 (41.4)	74 (57.4)	64 (52.5)
Right sided tumours, n (%)	189 (48.5)	69 (49.3)	63 (49.2)	57 (46.7)
Size (mm), median (IQR)	14 (8-22)	14 (8-22)	13 (9-23)	15 (8-22)
Size, n (%)				
Intrameatal	150 (38.5)	63 (45.3)	42 (32.6)	45 (36.9)
Small (1 to 10 mm)	235 (60.1)	90 (64.3)	73 (56.6)	72 (59.0)
Medium (up to 20 mm)	85 (21.7)	30 (21.4)	30 (23.3)	25 (20.5)
Large (up to 30 mm)	49 (12.5)	15 (10.7)	16 (12.4)	18 (14.8)
Very large (>30 mm)	22 (5.7)	5 (3.6)	10 (7.8)	7 (5.7)
Incidence*				
*Overall*	2.2	2.4	2.1	2.0
*By sex*				
Female	2.1	2.4	2.1	2.1
Male	2.2	2.8	0.9	1.9
*By age group*				
<40 years	0.5	0.7	0.3	0.4
40 – 49	2.1	2.1	2.1	2.2
50 – 59	3.8	4.2	3.6	3.5
60 – 69	5.8	5.7	6.1	5.8
>69	3.7	4.2	4.2	3.0
*By size*				
IM	0.8	1.1	0.7	0.7
Small (up to 10 mm)	1.3	1.5	1.2	1.2
Medium (>10 - 20 mm)	0.5	0.5	0.5	0.4
Moderately large (>20 - 30 mm)	0.3	0.1	0.3	0.3
Large (>30 mm)	0.1	0.1	0.2	0.1

*In the first column, incidence is expressed as the mean incidence per 100,000 person-years (overall, or in each population group by sex, age or tumour size) during the 3-year period. In the three last columns, incidence is expressed as number of cases per 100,000 population (overall or population group by sex, age or tumour size) per year

The mean annual incidence of newly diagnosed cases of VS was 2.2 per 100,000 person-years (Table 1). The annual estimated populations covered ranged from 5.9 to 6.1 million people. By sex, this mean incidence was 2.1 and 2.2 per 100,000 person-years for females and males respectively. By age group, mean incidence increased with age, from the <40 years age group to the 60–69 years age group, after which mean incidence decreased slightly (Table 1). The group with highest mean incidence was the 60–69 age group, with 5.8 cases per 100,000 person-years. By tumour size, mean incidence decreased with increasing tumour size, ranging from 0.1 per 100,000 person-years for large tumours to 1.3 cases per 100,000 person-years for small tumours IM tumour incidence was 0.8 per 100,000 person-years (Table 1).

**Fig. 1:**
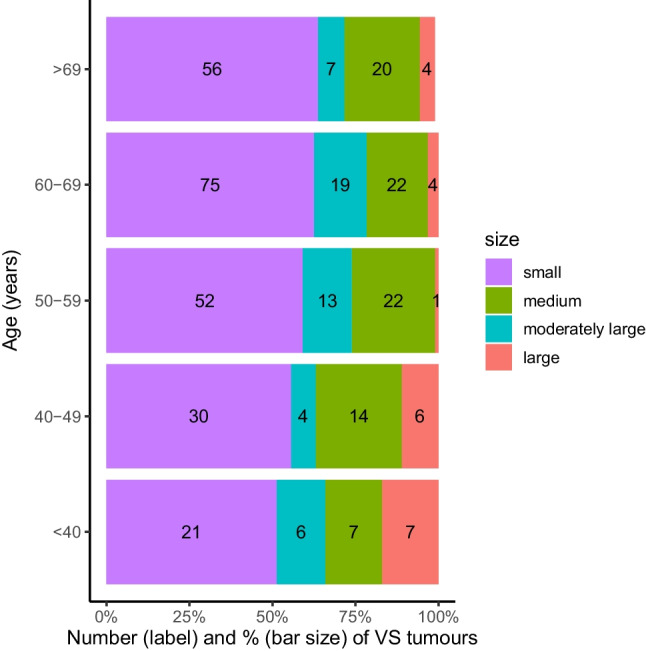
Number (label) and percentage (bar size) of newly diagnosed vestibular schwannoma tumours, by tumour size and age group

Among the 240 cases with EM tumours, the overall median (IQR) tumour size was 14 (4–44) mm. There was no statistically significant difference in the size of EM tumours by sex and year of diagnosis. Median (IQR) EM tumour size in female and male patients was 14 (6–40) mm and 13 (5–38) mm respectively. No statistically significant differences were identified in tumour size by sex (*p* = 0.93). However, age was inversely correlated with tumour size (r = -0.14 [-0.24 to -0.04], *p* = 0.01). Comparing across age groups, small tumours were the most common tumours in all age groups. Large tumours were more common among the youngest age groups (<40 and 40–49 years), as compared to the other age groups (Fig. 1).

### Symptom profile and duration

**Fig. 2:**
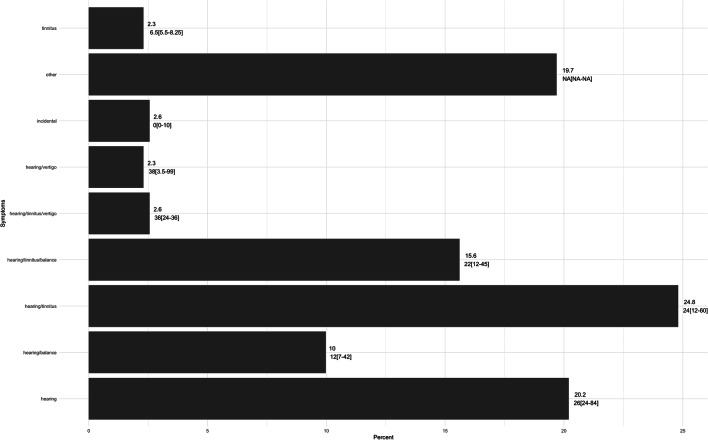
Percentage of patients presenting with each symptom profile, and median duration of each of symptom profile (*n* = 390). HL, hearing loss; IQR, interquartile range

EPR case-records were available for all cases except one. Hearing loss was the most common presenting symptom, being reported in nearly all diagnosed cases, either as the only symptom or combined with others (387 [99.2%]) (Fig. 2). In most of these, hearing loss was unilateral (373 [95.6%]). Hearing loss was followed by tinnitus (227 [58.2%]) and balance symptoms (169 [43.3%]), with vertigo (38 [9.7%]) and trigeminal (29 [7.4%]) symptoms being less common.

The top three combinations of symptoms on presentation per patient were hearing loss and tinnitus (97 [24.8%]), hearing loss alone (79 [20.2%]) and hearing loss, tinnitus, with balance symptoms (61 [15.6%]). Seventy-seven (19.7%) patients presented with other, atypical symptoms such as vertigo, bulbar symptoms and facial weakness. The remaining combinations of symptoms including, hearing loss and vertigo, hearing loss, tinnitus and vertigo and tinnitus alone were less common, reported by 28 (7.2%) of patients. Only 10 (2.6%) of patients were diagnosed incidentally (Figure 2).

**Fig. 3:**
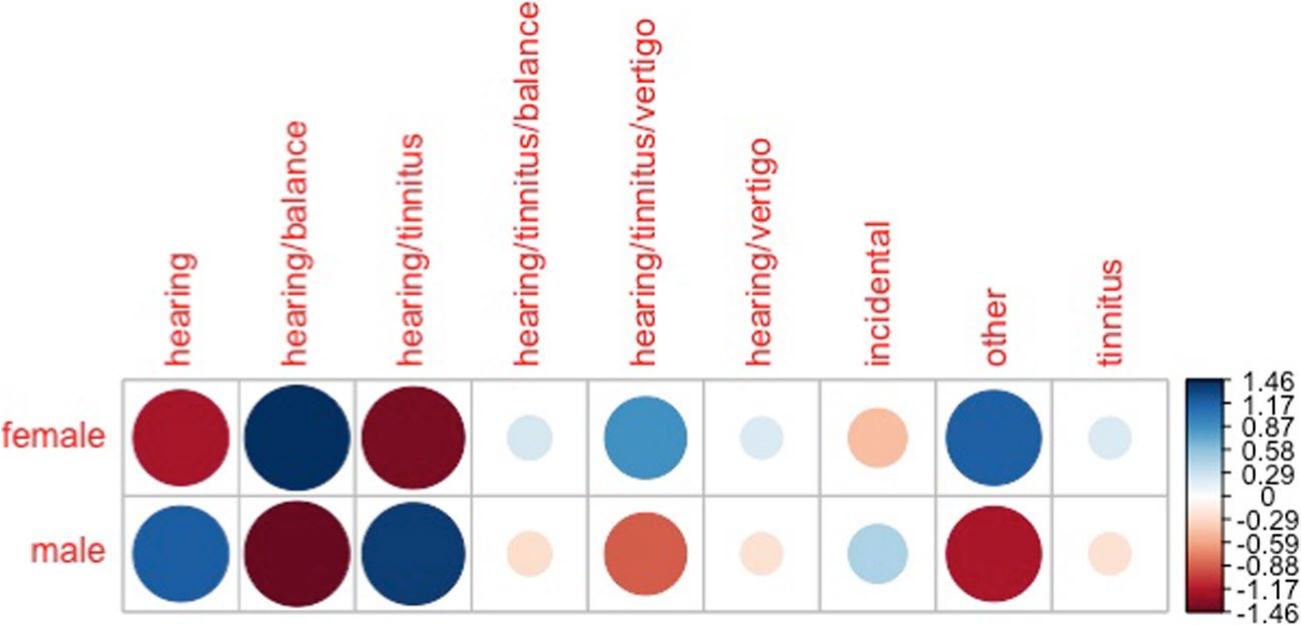
Correlation plot of Pearson residuals (standardised residuals) for each symptom profile and sex pair. The highest standardised residuals contribute the most to the chi-square test

The median age of patients reporting each symptom profile differed by age and sex. The median age was significantly different across different symptom profiles (*p* = 0.01). Post hoc Dunn’s test with Bonferroni correction showed that median age of patients presenting with hearing loss and tinnitus was significantly younger than patients presenting with hearing loss and balance symptoms (58 versus 67 years, corrected *p =* 0.02). Patients presenting with hearing loss and balance symptoms were also older than patients presenting with “Other” combinations of symptoms (67 versus 58 years, corrected *p* = 0.02). The proportion of patients reporting each symptom profile was significantly different by sex (*p* = 0.04). The plot of Pearson Residuals shows that male patients were strongly associated with reporting, (1) hearing loss and (2) hearing loss and tinnitus symptoms. Female patients were strongly associated with reporting, (1) hearing loss and balance and (2) hearing loss, tinnitus and vertigo. Female patients were also strongly associated with reporting rarer “Other” symptoms (Fig. 3).

In 97 (24.8%) cases, the duration of symptoms could not be ascertained from their case-records. Among those with a duration recorded, 230 had a numerical value recorded. Amongst these, the median (range) duration was 12 (0.1–25) years. Age was directly correlated with symptom duration (r = 0.16 [0.03–0.29], *p* = 0.02). Duration did not differ significantly by sex, or tumour size. (Fig. 2).

**Fig. 4:**
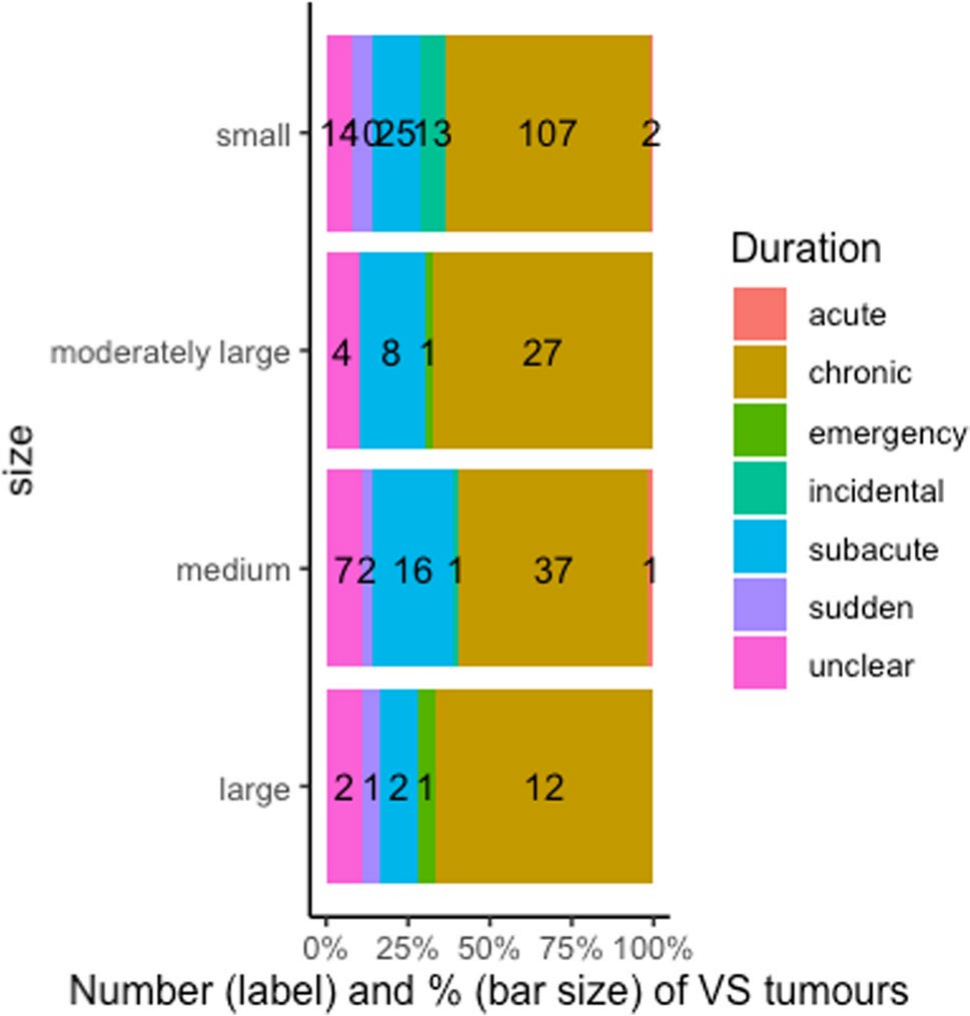
Number (label) and percentage (bar size) of presentation types (based on symptom duration) in each group of tumour size (*n* = 390). Tumour size classified in millimetres. Incidental: discovered after an unrelated investigation in asymptomatic patients; emergency: presented with hydrocephalus; sudden: with sudden onset of symptoms; acute: with symptoms for less than four weeks; subacute: with symptoms for four weeks to less than 12 months; chronic: with symptoms for 12 months or longer

Across all the age groups, most patients (184 [62.6%]) had chronic, or subacute symptoms (51 [17.4%]). Sudden (13 [4.4%]) and emergency (2 [0.7%]) presentations were uncommon (Fig. 4). Four to six cases per year (4.2–5.7%) were diagnosed incidentally following imaging for unrelated reasons and only two cases (2.1%) in 2014/15 presented as emergencies with hydrocephalus (Fig. 4).

By tumour size, (13 [92.8%]) of incidental diagnoses were for small tumours. The two cases diagnosed with hydrocephalus were moderately large and large tumours. Half of all small tumours (25 [49.1%]) presented with subacute symptoms. The proportion of patients with chronic symptoms increased as the tumour size increased (Fig. 4) but there was no significant correlation between tumour size and duration of symptoms (r = -0.03 [-0.17–0.11], *p* = 0.65).

## Discussion

Our study represents the most up-to-date report on VS epidemiology in the UK since 2002 [20].

We found a mean incidence of 2.2 new diagnoses of VS per 100,000 person-years over a 3-year period. Our study shows that the incidence of VS has increased compared to the period between 1990 and 1999 and the period between 1994 and 2002, where the reported incidence was estimated to be 0.8–1.4 per 100,000 person-years [5, 20]. Compared to international studies, our estimate of incidence lies in between estimates of overall incidence over the past decade which range between, 1.5 and 4.2 per 100,000 person-years in the period between 2012 and 2016 [10, 11, 15, 17, 22, 27].

The variation in reported incidence rates has been attributed to several factors including, differences in case ascertainment, demographic factors and health-system related factors [15]. Studies identifying cases from non-specific administrative registries have consistently reported lower incidence rates compared to prospectively maintained VS specific registries such as the Danish National Registry [10, 11, 19, 22, 27]. Systematic underreporting may be related to case mis-identification or variability in geographic reach between databases [16, 19]. Our study uses two independent data sources to identify cases of VS by cross-validating the UKSBR with linked hospital patient records increasing its reliability. This makes our study comparable to studies conducted in Denmark and Olmsted County, Minnesota, which are the only two other populations with prospective case identification where the incidence rates were reported to be between 3.3 and 4.2 per 100,000 person-years [17, 22].

Incidence did not differ by sex and peaked in the seventh decade for both female and male patients at a mean rate of 5.8 per 100,000 person-years. Small tumours (up to 10 mm) were the most common tumours diagnosed per year, particularly in older patients as there was a negative correlation between tumour size and age. Studies from Taiwan and Olmsted County have reported age-specific incidence rates showing that it peaks in the seventh decade between 4.8 and 9.3 per 100,000 person-years [11, 17]. The large variation in these reported rates is likely related to the aforementioned underreporting from administrative databases as the Taiwan study identified cases from a nationwide insurance database whereas similar to our study, in Olmsted County cases were identified through linked hospital records. Furthermore, unique demographical factors may contribute to the high detection rates in Olmsted County where healthcare contributes to 40% of the workforce [17]. Therefore, it is possible that the true incidence of VS peaks closer to 5.8–9.3 per 100,000 person-years. Overall, our findings support the hypothesis that increasing utilisation of MRI has led to the detection of more cases, in particularly of smaller tumours in older patients [10, 17, 22]. However, other studies have shown that despite the increasing incidence of MRI scans plateauing over time, the incidence of incidental cases of VS has not, suggesting that other contributary factors may be underlying the increase in VS incidence [18]. Nonetheless, there has been no strong evidence supporting any etiological factors such as noise exposure contributing to the increasing incidence rates [18]. In the current study, the rate of incidental cases of VS was uncommon at only 2.6%. This is tenfold lower then reported rates from Olmsted County where the rates are 24% in the last decade [15]. It is likely that this is due to differential health service conditions, given that by the age of 70 years, a third of people living in Olmsted County would have had a head MRI [18]. The UK has a state-funded healthcare system funded through general taxation similar to Denmark. Therefore, easier access to MRI services across populations may explain lower incidence rates in the UK compared to reported rates from Olmsted County and Denmark. In Olmsted County, patients are connected to a private healthcare service (Mayo Clinic Rochester) and in Denmark, every citizen can have access to an ENT Surgeon or have a MRI free-of charge without a referral, unlike in the UK which requires referrals from primary care providers [22]. This highlights the importance of considering local contextual factors when conducting epidemiological studies and the importance of international comparisons.

Another contributary factor to the increasing incidence found in our study relative to previous UK reports could be due to greater awareness of audiovestibular symptoms by patients leading to greater numbers of referrals from primary care providers. We found that the majority of patients presented with a history of hearing loss, tinnitus and balance symptoms. About 9 in 10 patients presented with unilateral hearing loss, about 6 in 10 with tinnitus and about 4 in 10 with balance problems. This is similar to symptoms reported by patients in Olmsted County [17]. This is the only other study which reported presenting symptoms in relation to incidence within a population. We report for the first time symptom profiles and duration of symptoms in relation to patient demographics. The most common mode of presentation was a long period (greater than 12 months) of hearing loss or hearing loss with either tinnitus and balance symptoms. Women were more likely to report hearing loss with balance or vertigo symptoms compared to men, as well reporting uncommon combinations (less than 5%) of “Other” symptoms such as headaches or trigeminal symptoms. Women have been reported to be 50% more likely to present with balance and dizziness symptoms [21]. The reasons for a sex difference in the prevalence of balance and trigeminal symptoms may be due to biological and social reasons [7, 23]. In other orofacial pain conditions such as trigeminal neuralgia, women have been reported to attend to somatic symptoms more frequently than men [23]. The median tumour size at presentation was 14 mm. Most patients presented with a long duration of symptoms (greater than 12 months). Half of small tumours presented with subacute (4 weeks to 12 months) but there was no significant correlation between tumour size and the symptom duration. Older patients were also more likely to present with longer duration of symptoms, particularly if they presented with hearing loss and tinnitus rather than with hearing loss and balance symptoms. Overall, our data shows that we are detecting small tumours in older symptomatic patients who already present with hearing loss. This raises several important issues, firstly, there should be greater awareness of hearing loss with tinnitus amongst primary care providers to reduce the time taken for diagnostic MRI and assessment by an ENT or neurosurgeon. This is crucial if designing screening studies since pure-tone audiometry has a lower sensitivity and specificity for detecting VS than MRI [6]. Secondly, greater numbers of small tumours diagnosed in older patients requires careful personalised management in order to preserve hearing. Sudden hearing loss can occur regardless of the size of tumour. We found that 4.4% of tumours presented with sudden HL. The causes of VS-induced HL can be related to microvascular causes, conduction block or endolymphatic hydrops, and these may not be related to tumour size [14]. This is consistent with our results as a similar proportion of tumours of different sizes presented with sudden symptoms. For patients with serviceable hearing at diagnosis, the rates of serviceable hearing reduce over time from 75 to 60% to 40% over 3-years, 5-years and 10-years [9]. This is comparable to patients treated with stereotactic radiosurgery where the probability of serviceable hearing at 2-years, 5-years and 10-years were, >75%, >50% and >25% respectively [4].

Our study findings may have been affected by a few limitations. We were not able to compare incidence rates in our catchment population with other populations in the UK as we did not have access to their case records. Therefore, the incidence rates in our population may differ from other UK populations due to geographical factors affecting diagnosis rates and access diagnostic imaging. To capture the full heterogeneity in incidence rates, future studies should compare both incidence rates within countries and internationally. In addition, we are also working on including access to presenting and follow-up imaging for patients in the UKVSR. This will allow future studies to investigate incidence with anatomical classification as well as size such as using Koos grading and correlate this with presenting symptoms [12]. Registry and retrospective record data can be affected by different types of bias, such as information bias. Assessments based on patient reported symptoms have the unavoidable risk of recall bias when patients are asked to recount their symptoms on diagnosis of VS. In addition, changes to the referral catchment areas can lead to changes in population size which can lead to variation in the calculated incidence rates. Nevertheless, the catchment area of the study centre did not change during the study period and incidence rates found in this study were stable across the 3-year period. Symptom duration could not be accurately ascertained from a quarter of the cases. This reduces the reliability of the symptom duration analysis. We do not believe this affects the overall conclusions showing the majority of the patients presenting with a long duration of symptoms as only a minority of patients present incidentally or with a short duration of symptoms.

A strength of our study is the inclusion, for the first time to our knowledge, of patient symptom profiles and durations based on clinician registered symptomatology in patient records during initial assessment. Another strength of this report includes case ascertainment through direct recording of referrals to skull base services for VS confirmed with imaging. This reduces the potential risk of bias derived from differences in coding practices in cancer registries, and under-reporting in surgical registries. This study uses clinician reported data, and diagnosis is based on gadolinium enhanced MRI, which is the gold-standard imaging test for the diagnosis of VS [14]. Furthermore, EPR review improved the accuracy of incidence estimates by excluding inaccurately coded cases which were diagnosed outside the study period. For example, certain cases may be diagnosed in the past and then re-referred to a physician when the patient is lost-to follow-up. However, EPR review excluded only 6% of cases, highlighting the overall reliability of the UKVSR data.

## Conclusion

Estimated incidence of vestibular schwannoma in the UK is 2.2 cases per 100,0000 person-years. Most patients are diagnosed with small tumours and present mainly with a combination of tinnitus, balance symptoms and hearing loss. Our findings can help inform the tailoring of health services for improving diagnosis and initial management of vestibular schwannoma.
